# Applications of Mendelian randomization in psychiatry: a comprehensive systematic review

**DOI:** 10.1097/YPG.0000000000000327

**Published:** 2022-10-20

**Authors:** Luigi F. Saccaro, Simone Gasparini, Grazia Rutigliano

**Affiliations:** a Department of Psychiatry, Faculty of Medicine, University of Geneva, Campus Biotech, Geneva, Switzerland; b Department of Psychiatry, Geneva University Hospital, Geneva, Switzerland; c Institute of Life Sciences, Sant’Anna School of Advanced Studies, Pisa, Italy; d Institute of Clinical Sciences, Faculty of Medicine, Imperial College London, London, UK

**Keywords:** ADHD, alcohol use disorder, autism spectrum disorder, bipolar disorder, depression, genetics, Mendelian randomization, psychiatry, schizophrenia, systematic review

## Abstract

Psychiatric diseases exact a heavy socioeconomic toll, and it is particularly difficult to identify their risk factors and causative mechanisms due to their multifactorial nature, the limited physiopathological insight, the many confounding factors, and the potential reverse causality between the risk factors and psychiatric diseases. These characteristics make Mendelian randomization (MR) a precious tool for studying these disorders. MR is an analytical method that employs genetic variants linked to a certain risk factor, to assess if an observational association between that risk factor and a health outcome is compatible with a causal relationship. We report the first systematic review of all existing applications and findings of MR in psychiatric disorders, aiming at facilitating the identification of risk factors that may be common to different psychiatric diseases, and paving the way to transdiagnostic MR studies in psychiatry, which are currently lacking. We searched Web of Knowledge, Scopus, and Pubmed databases (until 3 May 2022) for articles on MR in psychiatry. The protocol was preregistered in PROSPERO (CRD42021285647). We included methodological details and results from 50 articles, mainly on schizophrenia, major depression, autism spectrum disorders, and bipolar disorder. While this review shows how MR can offer unique opportunities for unraveling causal links in risk factors and etiological elements of specific psychiatric diseases and transdiagnostically, some methodological flaws in the existing literature limit reliability of results and probably underlie their heterogeneity. We highlight perspectives and recommendations for future works on MR in psychiatry.

## Introduction

**Fig. 1:**
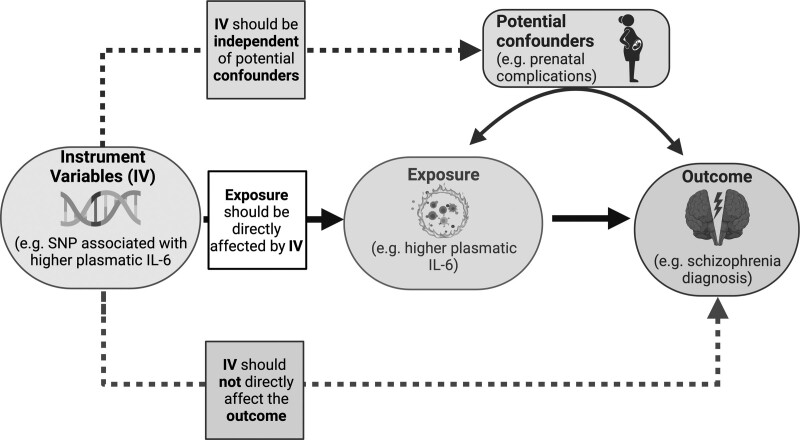
Diagram of a typical Mendelian randomization design, with, as an example, the key assumptions of a possible association between higher plasmatic IL-6 and schizophrenia risk. IL-6, Interleukin 6; IV, instrumental variable; SNP, single-nucleotide polymorphism.

Mental disorders comprehend a wide variety of diseases with different presentations. They are generally characterized by a combination of abnormal thoughts, perceptions, emotions, behavior, and relationships with others. Mental disorders include major depression disorder (MDD), bipolar disorder (BD), schizophrenia (SCZ) and other psychoses, and developmental disorders including autism spectrum disorders (ASD) or attention-deficit and hyperactivity disorder (ADHD). It is estimated that 14.3% of deaths worldwide are caused by mental disorders (for a total of approximately 8 million), which lead to more than 125 million disability-adjusted life-years (Walker *et al.*, 2015; GBD 2019 Mental Disorders Collaborators, 2019). Besides the heavy personal burden on patients and caregivers, psychiatric diseases also exact a heavy socioeconomic toll (Walker *et al.*, 2015). It is particularly difficult to identify risk factors and causative mechanisms for psychiatric diseases due to the limited physiopathological and etiological insight we have, to the many confounding factors, and to the potential reverse causality between the risk factors and these diseases, which are likely multifactorial (i.e. with important genetic and environmental risk factors). This is one of the reasons that make Mendelian randomization (MR) an especially precious tool for studying these diseases. MR is an analytical method that employs genetic variants [or instrumental variables (IVs)] linked to a certain risk factor, to assess if an observational association between that risk factor and a health outcome is compatible with a causal relationship between the risk factor and the chosen outcome. More in detail, every person is inherently assigned a genetic variant that could influence a risk factor in different ways [e.g. a variant that regulates the blood level of LDL could influence the risk of coronary artery disease (CAD)]. In a Mendelian randomized study, one or more of these genetic variants are followed up to verify the development of a specific health outcome. As recapitulated in Fig. 1, MR relies on three main assumptions: a relevance assumption (that the variant is associated with the risk factor, or exposure, in study), an exclusion restriction assumption (that the variants are affecting the outcome only through the risk factor/exposure), and an independence assumption (that these variants have not a shared cause with the outcome) (Emdin *et al.*, 2017).

Thus, it is clear the importance of the concept of pleiotropy, which is the production by one single genetic trait [such as a single-nucleotide polymorphism (SNP)] of two or more apparently unrelated outcomes. If pleiotropy arises because an SNP influences one trait (the exposure), which then influences another (i.e. the outcome), then vertical pleiotropy exists and MR can be used to estimate the causal relationship between exposure and outcome. Prominent among the limitations of MR studies, is the nondemonstrable assumption that the exposure mediates the apparent pleiotropic associations (thus reflecting vertical pleiotropy), and that the selected SNP does not influence both the exposure and the outcomes through unrelated pathways (thus violating the exclusion restriction assumption due to ‘horizontal pleiotropy’) (Hemani *et al.*, 2018). With these caveats, MR can use genetic associations obtained with genome-wide association studies (GWAS) to infer causality between exposures and outcomes.

Two main types of MR studies exist: the single sample randomization study and the double sample randomization study. The first one requires the measurement of the gene variant, the risk factor, and health outcome from the same sample of participants. The two-sample MR study requires two different study populations. In this approach, the gene variants and the health outcome are measured from one group, whereas the gene variants and risk factors are measured in another group (Pierce and Burgess, 2013). The main advantage of two-sample MR is that the outcome of interest and the risk factor do not need to be both measured in all participants, which is particularly important if they are expensive or difficult to measure. Thus, two-sample MR allows employing results from GWAS, which are usually precise and large studies (Davies *et al.*, 2018).

To recapitulate, MR is particularly relevant to study risk factors for diseases in which (a) it is difficult to ascertain causality between the risk factor and the disease, (b) reverse causality is possible, and (c) confounding factors are abundant and possibly hidden. Thus, thanks to its potentialities, MR is being used in different studies to evaluate the relationship between possible risk factors and the development of psychiatric diseases. As an example, MR may be employed to assess causality between BD risk and lifetime cannabis use (having ever used cannabis). In fact, if an association between cannabis use and BD were to be found with other study designs, it would be difficult to exclude that it is BD that leads to higher cannabis use, and not vice versa. Similarly, reverse causality is a substantial problem in SCZ psychoneuroimmunology, since associated psychological morbidities can lead to issues with personal hygiene or housing insecurity, which, together with medication side effects, can impact the immune system. Since MR uses genetic information (which generally existed before full-blown psychiatric symptoms) as risk factors, reverse causality is unlikely.

Important insights into MR limitations and strengths can emerge from literature reviews, as well as from the collaboration between clinicians, methodologists, and empirical researchers, from which have benefited also other areas of medical research (Davies *et al.*, 2018). While the interest of MR in psychiatry is increasingly prominent (Wootton *et al.*, 2022), most of the existing reviews focus on specific disorders (Belbasis *et al.*, 2018; Köhler *et al.*, 2018) or risk factors (Treur *et al.*, 2021), or they do not systematically review MR studies in psychiatry (Wootton *et al.*, 2022). In fact, to the best of our knowledge, there is no comprehensive overview of the applications of MR across different psychiatric diseases. Such a transdiagnostic approach would provide a unifying view of the limitations and potential of MR in psychiatry, as well as stimulating insight into potential common risk factors for different psychiatric disorders.

With the present systematic review, we thus aim at providing, for the first time, an unbiased and inclusive view of all existing applications of MR in psychiatric disorders, as defined by Diagnostic and Statistical Manual of Mental Disorders (DSM) or International Classification of Diseases (ICD) diagnoses. Such an approach aims at facilitating the identification of risk factors that may be common to different psychiatric diseases, and at paving the way to transdiagnostic MR studies in psychiatry (which are currently under-investigated), in agreement with current psychiatry research models advising to target aspects common to different pathologies, rather than traditionally defined diagnoses only (Insel, 2014).

## Methods

### Search strategy and selection criteria

This systematic review was conducted following the Preferred Reporting Items for Systematic Reviews and Meta-analyses (PRISMA) guidelines (Supplementary eTable 1, Supplemental Digital Content 1, http://links.lww.com/PG/A287) (Moher *et al.*, 2009), and is registered in the PROSPERO database (registration number CRD42021285647).

We used a two-step approach. First, we searched the Web of Science database by Thomas Reuters, Pubmed, and Scopus. The search strategy included terms related to MR and psychiatry [((Mendelian randomization) OR (Mendelian randomisation)) AND (psychiatry OR bipolar disorder OR borderline personality disorder OR schizophrenia OR depression OR ADHD OR anxiety OR PTSD OR panic))] for articles published until 3 May 2022. We excluded reviews or metanalysis through the search filters. Second, we performed a manual search of the lists of references of retrieved articles. Duplicate references were manually excluded. The remaining articles were screened by title and abstract, and the full texts identified were further inspected for eligibility against *a priori*-defined exclusion and inclusion criteria. We included original articles in English that met the following Participants, Interventions, Comparators, Outcomes, and Study design criteria. Participants of the study were psychiatric patients of any age, with any psychiatric diagnosis according to DSM or ICD criteria. The Intervention had to include the employment of MR in genetic analysis, applied to the identification of risk factors, to neuroimaging correlations, or to other settings relating to psychiatry (including studies that focused on how psychiatric diseases might be risk factors for other conditions). Comparators were the presence or absence of genetic traits that are determinants of exposure to a certain risk factor (which could be a psychiatric disorder or other risk factors). The Outcome was the risk for a certain condition (both psychiatric and not).

**Fig. 2:**
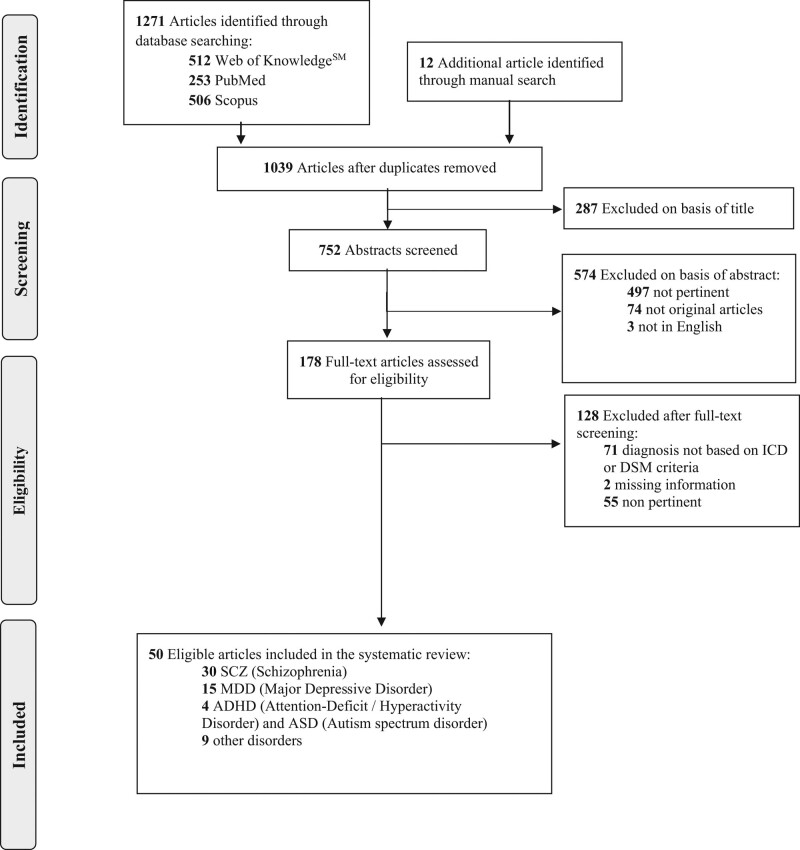
PRISMA 2009 flow diagram. PRISMA, Preferred Reporting Items for Systematic Reviews and Meta-analyses.

We included all study designs apart from case reports, case series, conference abstracts and presentations, pilot/feasibility studies, reviews, meta-analyses, and systematic reviews. Of note, we did not exclude studies that analyzed (considering different exposures/outcomes, or using different methods from each other) data stored in publicly available databases, or overlapping or partly overlapping populations. The selection process was documented in the PRISMA flow diagram (Fig. 2).

### Data extraction

Data extraction was performed by independent researchers (L.F.S. and S.G.). Any discrepancy was discussed until a consensus was reached. Disagreements were resolved by a third reviewer (G.R.).

The following variables were extracted from each article: authors, year of publication, sample size, genetic information analyzed, main MR method, main findings, presence of pleiotropy analysis, and psychiatric disorder considered as an outcome. If more than one method were employed and results were homogeneous across methods, we reported the first one described. If results obtained through different methods of analysis differed, we reported each method in the corresponding column, and we indicated between parathesis which method was employed to obtain each result in the ‘Outcome and finding’ column. If variables were not available, and no reply was obtained from the corresponding author of the article concerned (at least a 1-month delay for each query), we wrote ‘NG’, not given. If crucial information was missing, we contacted the corresponding author and excluded the study if no answer was received (at least 1-month delay for each query). In the case of articles reporting on more than one psychiatric disease, we included only results on diseases that had been diagnosed according to ICD or DSM criteria, in agreement with our inclusion criteria.

### Quality assessment

The quality of the selected studies was assessed independently by two reviewers (S.G. and L.F.S.) with the Newcastle-Ottawa Scale (NOS) (Lo *et al.*, 2014). Any discrepancy was discussed until a consensus was reached. Disagreements were resolved by a third reviewer (G.R.). The NOS is a risk of bias assessment tool that has been used also to evaluate MR studies (Cheng *et al.*, 2020; Spiga *et al.*, 2021). It consists of two sections: one for case-report studies and the other for cohort studies. Studies were evaluated using NOS considering three aspects: patient selection, comparability, and exposure (Supplementary eTable 2, Supplemental Digital Content 1, http://links.lww.com/PG/A287). The risk of bias and concerns regarding applicability were analyzed for each domain.

## Results

As described in Fig. 2, 1039 nonduplicates studies were selected through database searching. Two hundred and eighty-seven of these were excluded based on the title, as they were not relevant. A total of 752 articles were screened based on the abstract to exclude articles that were not in English (*n* = 3), that were not pertinent (i.e. not about MR in psychiatry) (*n* = 497) or not original articles (e.g. reviews, case reports, metanalyses, and opinion articles) (*n* = 74). Upon eligibility screening, 128 studies were not eligible as 71 articles included at least some psychiatric patients with diagnoses not based on ICD or DSM criteria, two studies had missing information (and no reply was obtained from the corresponding author of the article concerned after at least 1-month delay for each query), and 55 articles were not pertinent.

The final sample, thus, included 50 articles. Although we searched for studies published from 1966 until 2022, the oldest included study dates back to 2014 (Hung *et al.*, 2014). This confirms the novelty of this research topic. Thirty studies focused on SCZ, 15 on MDD, four on ADHD or ASD, and nine on other disorders. Of note, the sum of the articles included in each group does not equal 50 because studies presenting distinct results concerning multiple diseases are reported in each of the relevant tables for each disease. Details of each study are reported in Tables 1–4.

The NOS scores were fair and homogenous among the studies. More in detail, all studies scored high in the ‘selections of patients’ and ‘comparability’. Further details are provided in Supplementary eTable 2, Supplemental Digital Content 1, http://links.lww.com/PG/A287.

### Mendelian randomization in schizophrenia

The most numerous group of articles that we included is on SCZ, comprising 30 articles (Table 1) (Wium-Andersen *et al*., 2015; Prins *et al.*, 2016; Taylor *et al.*, 2016; Inoshita *et al.*, 2016; Gage *et al.*, 2017a; Gage *et al.*, 2017b; Hartwig *et al.*, 2017; Arafat and Minica, 2018; Li *et al.*, 2018; Pasman *et al.*, 2018; Polimanti *et al.*, 2018; Tomioka *et al.*, 2018; Byrne *et al.*, 2019; Gao *et al.*, 2019; Jang *et al.*, 2020; Jones *et al.*, 2020; Kim et al., 2020, 2021; Luo *et al.*, 2020; Peters *et al.*, 2020; Wootton *et al.*, 2020; Yang *et al.*, 2020; Zhuang *et al.*, 2020; Jin *et al.*, 2021; Johnson *et al.*, 2021; Perry *et al.*, 2021b; Song *et al.*, 2021; Chen et al., 2021, 2022; Andreu-Bernabeu *et al.*, 2022; Ni *et al.*, 2022). Most studies focused on inflammation, metabolic traits, or substance use and their associations with SCZ.

**Table 1:** Mendelian randomization in schizophrenia

References	Genetic instrument and exposure (*P*-value)	Main statistic test	Exclusion of pleiotropy	Outcome and findings	Outcome sample size
Andreu-Bernabeu *et al.*, 2022	116 SNPs associated with SCZ (*P* < 5 × 10^−8^)	WM	Yes	Bidirectional causal effect between polygenic scores for loneliness/isolation and SCZ risk (WM−β (SE) = 1.37 (0.40); *P* = 6.14 × 10−4)	35 476 SCZ cases
	19 SNPs associated with loneliness/social isolation (*P* < 5 × 10^−6^)				46 839 controls (PGC)
Chen *et al.*, 2022	2422 SNPs associated with 41 systemic inflammatory regulators, 2788 SNPs associated with SCZ (*P* < 1 × 10^−6^)	IVW	Yes	Genetic predisposition to increased HGF, IL-17, IL-1ra, MCP3, TRAIL causally associated with higher SCZ risk (*P* < 5 × 10^−2^)	36 989 SCZ cases
				Genetic liability to SCZ causally associated with CTAK and RANTES (*P* < 3 × 10^−2^)	11 3075 controls (PGC)
Ni *et al.*, 2022	>700 SNPs associated with gut microbiota and 28 SNPs associated with SCZ (*P* < 1 × 10^−5^)	IVW	Yes	Genetic predisposition to increased class Actinobacteria causally associated with higher SCZ risk (*P* = 1.33 × 10^−3^)	152 805 SCZ cases, 18 473 controls (MiBioGen)
				No causal association between genetic instruments for SCZ and risk of different gut microbiota composition	
Perry *et al.*, 2021b	53 SNP associated with insulin resistance phenotype (*P* < 5 × 10^−3^)	IVW	Yes	No causal association between genetic instruments for insulin resistance and SCZ (IVW)	40 675 SCZ cases
	58 SNP associated with CRP (*P* < 5 × 10^−8^)	MVMR		No significant association between inflammation-related SNP and SCZ after adjusting for genetic instruments for CRP (MVMR)	64 643 controls
Kim *et al.*, 2021	4 SNP associated with PD risk (*P* < 5 × 10^−6^)	IVW	Yes	Genetic predisposition to PD causally associated with higher SCZ risk (OR, 1.10; 95% CI, 1.05−1.15; *P* = 3.49 × 10^−5^)	35 476 SCZ cases
					46 839 controls (PGC)
Jin *et al.*, 2021	93 SNP associated with PCOS (*P* < 5 × 10^−8^)	IVW,	Yes	No causal association between genetic instruments for PCOS predisposition and SCZ risk	33 640 SCZ cases
					43 456 controls
Chen *et al.*, 2021	31 SNP associated with cigarette smoking (*P* < 1 × 10^−5^)	IVW, MR-Egger	Yes	No causal association between genetic instruments for smoking and SCZ	7711 SCZ cases
					18 327 controls
Yang *et al.*, 2020	530 SNP associated with 2-methoxyacetaminophen sulfate levels (*P* < 1 × 10^−5^)	IVW	Yes	Genetic predisposition to higher 2-methoxyacetaminophen sulfate level causally associated with higher SCZ risk (*P* = 1.73 × 10^−5^)	36 989 SCZ cases and 113 075 controls
Pasman *et al.*, 2018	74 SNP associated with cannabis use (*P* < 1 × 10^−5^)	IVW	Yes	Genetic predisposition to cannabis use causally associated with higher SCZ risk (OR, 1.10; 95% CI, 0.99–1.21; *P* = 0.074)	130 072 CZ cases
	109 with SCZ (*P* < 5 × 10^−8^)			Genetic liability to SCZ causally associated with cannabis use (OR, 1.16; 95% CI, 1.06–1.27; *P* = 0.001)	180 934 cases of cannabis use
Zhuang *et al.*, 2020	30 SNP associated with Enterobacteriaceae family and order (*P* = 3.72 × 10^−11^)	IVW	Yes	Genetic predisposition to increased:	21 246 SCZ cases
				Enterobacteriaceae family and order causally associated with higher SCZ risk (OR, 1.09; CI, 1.00–1.18; *P* = 0.048)	
				Gammaproteobacteria class (OR, 0.90; CI, 0.83–0.98; *P* = 0.011) causally associated with lower SCZ risk	
	15 with Gammaproteobacteria class (*P* < 1.4 × 10^−8^)			Gut production of serotonin causally associated with higher SCZ risk (OR, 1.07; CI, 1.00–1.15; *P* = 0.047)	
	32 with increased gut production of serotonin (*P* < 8.96 × 10^−6^)			No causal associations with SCZ risk for other types of gut microbiota	38 072 controls
Wootton *et al.*, 2020	114 SNP associated with SCZ	IVW	Yes	Genetic predisposition to lifetime smoking causally associated with higher SCZ risk (OR, 2.27; 95% CI, 1.67–3.08; *P* < 0.001)	36 989 SCZ cases
	126 with lifetime smoking (*P* < 5 × 10^−8^)			Genetic liability for SCZ causally associated with higher risk of lifetime smoking (β= 0.022; 95% CI, 0.005–0.038; *P* = 0.009)	113 075 controls (PGC)
					213 372 ever-smokers and 249 318 never-smokers (UK Biobank)
Peters *et al.*, 2020	319 SNP associated with WHR_adjBMI_	IVW, MR-Egger	Yes	No causal association between genetic instruments for WHR_adjBMI_ and SCZ risk	40 675 SCZ cases
					64 643 controls (PGC)
Kim *et al.*, 2020	70 SNP associated with SCZ (*P* < 5 × 10^−8^)	IVW	Yes	Genetic liability to SCZ causally associated with higher breast cancer risk (OR, per log odds increase in SCZ risk, 1.069; 95% CI, 1.028–1.112; *P* < 0.001)	82 315 SCZ cases (PGC)
	117, 146, 204 SNP associated with breast cancer (5 × 10^−8^, 5 × 10^−7^, 5 × 10^−6^respectively)			No causal association between genetic instruments for breast cancer and SCZ	228 951 breast cancer cases (BCAC)
Jones *et al.*, 2020	5 SNP associated with anxiety	IVW	Yes	Genetic liability to neuroticism (OR, 1.33; 95% CI, 1.12–1.59) and anxiety (OR, 1.10; 95% CI, 1.01–1.19) causally associated with higher SCZ risk	33 640 SCZ cases
	116 with neuroticism (*P* < 5 × 10^−8^)				43 456 controls (PGC)
Jang *et al.*, 2020	341 SNP associated with substance use phenotypes (smoking, alcohol, or cannabis use)	IVW, WM, MR-Egger; Latent Causal Variable model	Yes	No causal association between genetic instruments for substance use phenotypes and the risk SCZ	105 318 SCZ cases
Song *et al.*, 2021	1 SNP associated with pars triangularis volume (*P* < 5 × 10^−8^)	IVW	No	Genetic predisposition to lower pars triangularis volume causally associated with SCZ risk (OR, 0.48; 95% CI, 0.34-0.69; *P* = 5.9 × 10^−5^)	40 675 SCZ cases
					64 643 controls (PGC)
Luo *et al.*, 2020	45 SNP associated with SCZ (*P* < 5 × 10^−8^)	IVW	Yes	Genetic liability to SCZ causally associated with higher serum uric acid risk (per 10-s% increase in SCZ risk: beta: −0.039, SE: 0.013; *P* = 0.003)	35 476 SCZ cases
	26 SNP associated with serum uric acid (*P* < 5 × 10^−8^)			No causal association between genetic instruments for higher serum uric acid and SCZ	46 839 controls (PGC)
Gao *et al.*, 2019	110 SNP associated with insomnia (*P* < 5 × 10^−8^)	MR-Egger, GSMR	Yes	No causal association between genetic instruments for insomnia and SCZ	33 426 SCZ cases
					32 541 controls
Byrne *et al.*, 2019	142 SNP associated with SCZ	GSMR	Yes	Genetic liability to SCZ causally associated with higher breast cancer risk (SE = 0.008; *P* = 2.2 × 10^−4^)	40 675 SCZ cases and 64 643 controls (PGC)
	191 with breast cancer (*P* < 5 × 10^−8^)			No causal association between genetic instruments for breast cancer and SCZ	122 977 breast cancer cases and 105 974 controls
Tomioka *et al.*, 2018	1 SNP associated with serum pyridoxal levels (*P* = 0.006)	IVW	No	No causal association between genetic instruments for serum pyridoxal level and SCZ	365 SCZ cases
					911 controls
Polimanti *et al.* 2018	10 SNP associated with T2D (*P* < 5 × 10^−8^)	Instrumental variable analysis	Yes	No bidirectional causal associations between SCZ and T2D	34 840 T2D cases and 114 981 controls (DIAGRAM consortium)
	108 SNP associated with SCZ (*P* < 5 × 10^−8^)				34 241 SCZ cases and 45 604 controls (PGC)
Arafat and Minica, 2018	60 SNP associated with birth weight (*P* < 5 × 10^−8^)	IVW	Yes	No causal association between genetic instruments for birth weight and SCZ	34 241 SCZ cases
					45 604 controls (PGC)
Li *et al.*, 2018	13 SNP associated with fasting insulin levels	IVW	Yes	No causal effect of any of the genetic instruments on SCZ risk, nor in Europeans nor in East Asians, in the BMI-adjusted analysis	84 514 SCZ cases
	30 with fasting glucose in Europeans, 14 in East Asians				126 949 controls (PGC2, BIOX)
	36 with HbA1c in Europeans, 27 in East Asians				
	140 with T2D (for Europeans, East Asians and trans-ancestry groups) (*P* < 5 × 10^−8^)				
Hartwig *et al.*, 2017	18 SNP associated with CRP serum levels	IVW	Yes	Genetic predisposition to higher serum CRP causally associated with lower SCZ risk (OR, 0.90; 95% CI, 0.84-0.97; *P* = 0.005)	36 989 SCZ cases
	1 associated with higher sIL-6R serum levels			Genetic predisposition to higher serum sIL-6R causally associated with higher SCZ risk (OR, 1.06; 95% CI, 1.01-1.12; *P* = 0.02)	
	2 associated with higher IL-1Ra serum levels (*P* < 5 × 10^−5^)			No causal association between genetic instruments for IL-1Ra and SCZ risk	113 075 controls (PGC)
Gage *et al.*, 2017a	128 SNP associated with SCZ risk (*P* < 5 × 10^−8^)	IVW	Yes	Genetic liability to SCZ causally associated with risk for cannabis initiation (OR, 1.10 per doubling of the odds of SCZ; 95% CI, 1.05–1.14; *P* = 2.64 × 10^−5^)	36 989 SCZ cases
	21 with cannabis initiation (*P* < 10^−5^)			Genetic predisposition to cannabis initiation causally associated with SCZ risk (OR, 1.04 per doubling odds of cannabis initiation; 95% CI, 1.01–1.07; *P* = 0.019)	113 075 controls (PGC)
Gage *et al.*, 2017b	21 SNP associated with smoking initiation (*P* < 10^−6^)	IVW	Yes	Genetic predisposition to smoking initiation causally associated with SCZ risk (OR, 1.73; 95% CI, 1.30–2.25, *P* < 0.001)	36 989 SCZ cases
	94 with SCZ risk (*P* < 5 × 10^−8^)			No causal association between genetic instruments for SCZ and risk for smoking initiation (OR, 1.01; 95% CI, 0.98–1.04; *P* = 0.32).	113 075 controls (PGC)
					35 845 smokers (TAG consortium)
Taylor *et al.*, 2016	4 SNP associated with serum vitamin D levels (*P* < 6 × 10^−10)^	IVW	Yes	No causal association between genetic instruments for higher serum vitamin D and SCZ risk.	36 989 SCZ cases
					113 075 controls (PGC)
Prins *et al.*, 2016	18 SNP associated with CRP levels (*P* < 1 × 10^−4^)	GRS IV	Yes	Genetic predisposition to higher serum CRP causally associated with reduced SCZ risk (per 10-s% increase in CRP level; OR, 0.86; 95% CI, 0.79–0.94; *P* < 0.0010)	34 241 SCZ cases
					45 604 controls (PGC)
Inoshita *et al.*, 2016	15 SNP associated with CRP serum levels (*P* < 5 × 10^−8^)	IVW	Yes	Genetic predisposition to higher serum CRP causally associated with SCZ risk (OR, 1.10; 95% CI, 1.02–1.19; *P* = 0.015)	418 SCZ cases
					1365 controls
Wium-Andersen *et al.*, 2015a	1 SNP associated with smoking intensity	IVW	No	Genetic predisposition to higher smoking intensity causally associated with SCZ risk (OR, 1.06; 95% CI, 1.04–1.08) in ever- and never-smokers combined, but not in each group alone.	67 SCZ cases
					40 014 ever-smokers and 23 282 never-smokers

BCAC, Breast Cancer Association Consortium; BIOX, Bio-X Institutes; CI, confidence interval; CRP, C-reactive protein; CUD, cannabis use disorder; HGF, hepatocyte growth factor; CTACK, cutaneous T-cell attracting chemokine; GRS IV, Genetic risk score instrumental variable; (GS)MR, (Generalized Summary-based) Mendelian randomization; (s)IL-R, (soluble) interleukin receptor; IVW, inverse variance–weighted; MCP3, monocyte-specific chemokine 3; MVMR, multivariable Mendelian randomization; OR, odds ration; PGC, psychiatric genomic consortium; RANTES, regulated on Activation; SCZ, schizophrenia; SE, standard error; SNP, single-nucleotide polymorphisms; s%, symmetric percentage; TAG, Tobacco and Genetics Consortium; TRAIL, TNF-related apoptosis-inducing ligand; T2D, type 2 diabetes; WHR_adjBMI_, waist-to-hip ratio adjusted for BMI; WM, weighted median.

Contradictory findings exist on the association between smoking and risk for SCZ: while Chen *et al.* (2021) did not find any causal association between their genetic instrument for smoking and SCZ risk, two other studies with more numerous sample sizes did show that genetic predisposition to smoking was causally associated with higher SCZ risk, but not vice versa (Gage *et al.*, 2017b; Wootton *et al.*, 2020). On the other hand, there may be a bidirectional causal association between cannabis use and SCZ that is genetic risk for SCZ is causally associated with cannabis use and vice versa (Gage *et al.*, 2017a; Pasman *et al.*, 2018).

Metabolic syndrome is a well-known comorbidity of patients with SCZ, but this is likely due to antipsychotic use. In fact, no causal relationship has been highlighted between genetic instruments for metabolic traits [e.g. insulin resistance (Li *et al.*, 2018; Perry *et al.*, 2021b), BMI (Peters *et al.*, 2020), or diabetes (Polimanti *et al.*, 2018)] and SCZ risk.

Findings on the link between the immune system and SCZ risk are contrasting (Prins *et al.*, 2016; Hartwig *et al.*, 2017; Chen *et al.*, 2022). Two papers studying partially overlapping samples from the Psychiatric Genomics Consortium (PGC) highlighted that a genetic predisposition for a higher serum C-reactive protein (CRP) was causally associated with reduced SCZ risk (Prins *et al.*, 2016; Hartwig *et al.*, 2017), whereas a study on a smaller sample size reported opposite findings (Inoshita *et al.*, 2016). Studying systemic inflammation in SCZ is particularly relevant considering that two recent studies showed causal links between genetic predisposition to altered gut microbiota composition and SCZ risk (Zhuang *et al.*, 2020; Ni *et al.*, 2022), and it is known that microbiota composition might influence systemic immunity (Lo *et al.*, 2021). However, the classes of gut bacteria identified as risk factors for SCZ differ between these two studies [i.e. actinobacteria by Ni *et al*. (2022) and gammaproteobacteria by Zhuang *et al.* (2020)].

Interestingly, genetic liability to other psychiatric disorders (i.e. anxiety and neuroticism) was causally associated with higher SCZ risk (Jones *et al.*, 2020), whereas this was not true for insomnia (Gao *et al.*, 2019).

Finally, two works (Byrne *et al.*, 2019; Kim *et al.*, 2020) found that genetic liability to SCZ was causally associated with an increased risk for developing breast cancer (but not vice versa), in partially overlapping populations.

### Mendelian randomization in major depressive disorder

We included 15 studies on MR in MDD (Table 2) including data from populations ranging between 500 and 143 265 MDD patients (Wium-Andersen *et al.*, 2014; Hung *et al.*, 2014; Wium-Andersen *et al.*, 2015a; Wium-Andersen *et al.*, 2015b; Kwok *et al.*, 2016; Sequeira *et al.*, 2017; Clarke *et al.*, 2017; Michalek *et al.*, 2017; Cai *et al.*, 2019; Choi *et al.*, 2019; Milaneschi *et al.*, 2019; Gill *et al.*, 2019; Huang *et al.*, 2020; Lu *et al.*, 2021; Maharjan *et al.*, 2021). Studies employed most often inverse variance–weighted analysis, and they all accounted for pleiotropic effects, apart from (Michalek *et al.*, 2017). These mostly evaluated causal associations between exposure to metabolic traits, or to other diseases (such as CAD or stroke) and MDD. MDD was, thus, used as exposure and/or outcome. For instance, genetic variables for diabetes (Clarke *et al.*, 2017), higher plasmatic CRP (Wium-Andersen *et al.*, 2014), vitamin D or n3-PUFA (Milaneschi *et al.*, 2019) or BMI (Hung *et al.*, 2014) did not lead to an increased risk for MDD. While no bidirectional causal association between genetic instruments for Alzheimer’s disease and MDD was identified (Huang *et al.*, 2020), genetic liability to MDD appears to impair functional outcome after a stroke (Gill *et al.*, 2019), and to be causally associated with cardiovascular disease (Lu *et al.*, 2021), as well as with higher risk of small vessel stroke, although this effect is not present for large artery nor cardioembolic strokes (Cai *et al.*, 2019).

**Table 2:** Mendelian randomization in major depressive disorder

Reference	Genetic instrument and exposure (*P*-value)	Main statistic test	Exclusion of pleiotropy	Outcome and findings	Outcome sample size
Maharjan *et al.*, 2021	121 SNP associated with total testosterone	IVW	Yes	No causal association between genetic instruments for increased female testosterone or SHBG levels and MDD	521 MDD cases
	91 with bioavailable testosterone				
	173 with serum SHBG protein				
					368 controls
Lu *et al.*, 2021	102 SNP associated with MDD (*P* < 5 × 10^−8^)	IVW	Yes	Genetic liability to MDD causally associated with higher CAD (OR, 1.14; 95% CI, 1.06–1.24; *P* = 1.0 × 10^−3^) and myocardial infarction (OR, 1.21; 95% CI, 1.11–1.33; *P* = 4.8 × 10^−5^) risks	60 801 CAD cases, including 43 676 with myocardial infarction,
					123 504 controls
Huang *et al.*, 2020	32 SNP associated with Alzheimer’s Disease, and 65 SNP with MDD (*P* < 0.002)	IVW	Yes	No bidirectional causal association between Alzheimer’s Disease and MDD genetic instruments	9240 MDD cases
					9519 controls
Milaneschi *et al.*, 2019	6 SNP associated with vitamin D	IVW	Yes	No bidirectional causal association between vitamin D or n3-PUFA and MDD genetic instruments	1700 MDD cases
					347controls
	7 with n3-PUFA				
	37 with MDD (*P* < 0.05)				
Gill *et al.*, 2019	56 SNP associated with MDD (*P* < 5 × 10-8)	IVW	Yes	Genetic liability to MDD causally associated with functional outcome after ischemic stroke (OR of poor outcome per 1-SD increase in genetically determined risk of MDD, 1.81; 95% CI, 0.98–3.35; *P* = 0.06).	60 341 ischemic stroke cases
					454 450 control subjects (MEGASTROKE consortium)
Cai *et al.*, 2019	72 SNP associated with MDD (*P* < 1 × 10^−6)^	IVW	Yes	Genetic liability to MDD:	34 217 ischemic stroke cases
				causally associated with higher risk of small vessel stroke, (OR, 1.33; 95% CI, 1.08–1.65; *P* = 0.009)	
					406 111 controls
				but not with large artery stroke, cardioembolic stroke, or all ischemic stroke	
Choi *et al.*, 2019	SNP associated with:	IVW	Yes	Genetic predisposition to higher accelerometer-based physical activity causally associated with reduced risk for MDD (IVW OR, 0.74 for MDD per 1-SD unit increase in mean acceleration; 95% CI, 0.59-0.92; *P* = 0.006)	143 265 MDD
	MDD (*n* = 17) (*P* < 1 × 10^−6^)				91 084 accelerometer-based activity
	Accelerometer-based (*n* = 12) and self-reported (*n* = 25) physical activity (*P* < 1 × 10^−7^)				
					377 234 self-reported activity
				No causal association between genetic instruments for accelerometer-based activity and MDD	
				No causal association between genetic instruments for self-reported activity and MDD	
Michalek *et al.*, 2017	1 SNP associated with shorter leukocyte telomere lengths	Generalized linear model	NO	Genetic predisposition to shorter telomere lengths causally associated with:	1628 MDD cases
					1140 controls
				Increased risk for childhood-onset MDD (*P* ≤ 0.05)	
				Increased risk for childhood-onset MDD relative to adult-onset MDD cases (*P* ≤ 0.001)	
				No association with adult-onset MDD	
Wium-Andersen *et al.*, 2014	4 SNP associated with increased plasma CRP levels (*P* < 5.1 × 10^−59^)	Regression model	Yes	No causal association between genetic instruments for increased plasma CRP levels and MDD	1183 MDD
					77 626 controls
Sequeira *et al.*, 2017	120 SNP associated with age at menarche (*P* < 0.0001)	Structural mean model	Yes	Genetic liability to early menarche:	3920 girls (ALSPAC cohort)
				Causally associated with higher levels of depressive symptoms at 14 years (OR, per risk allele 1.02; 95% CI, 1.005–1.04, *n* = 2404).	
				No association with MDD after 14 years	
Kwok *et al.*, 2016	3 SNP associated with coffee consumption (*P* < 5 × 10^−8^)	IVW	Yes	No causal association between genetic instruments for coffee consumption and MDD	9240 MDD cases
					9519 controls (PGC)
Clarke *et al.*, 2017	10 SNP associated with T2D (*P* ≤ 5 × 10^−8^)	Mixed linear models	Yes	No causal association between genetic instruments for T2D and MDD	2697 MDD cases
					20 800 controls (GS: SFHS)
Hung *et al.*, 2014	32 SNP associated with higher BMI (*P* < 5 × 10^−8^)	Maximum likelihood estimation model	Yes	No causal association between genetic instruments for higher BMI and MDD	2430 MDD cases
					792 controls
Wium-Andersen *et al.*, 2015b	1 SNP associated with smoking intensity	IVW	No	No causal association between genetic instruments for higher smoking intensity and MDD risk in any group	1067 MDD cases
					40 014 ever-smokers and 23 282 never-smokers
Wium-Andersen *et al.*, 2015a	2 SNP associated with alcohol consumption (*P* < 1 × 10^−5^)	IVW	No	Genetic predisposition to alcohol consumption causally associated with higher risk for hospitalization/death with MDD (OR, 4.52; 95% CI, 0.99–20.5; *P* = NG)	4777 cases of hospitalization/death with MDD
					78 154 total participants

Longitudinal Study of Parents and Children; CAD, coronary artery disease; CI, confidence interval; GS: SFHS, Generation Scotland: the Scottish Family Health Study; IVW, inverse variance–weighted; MDD, major depressive disorder; n3-PUFA, omega-3 polyunsaturated fatty acid; NG, not given; OR, odds ration; PGC, psychiatric genomic consortium; SHBG, sex-hormone-binding globulin; SNP, single-nucleotide polymorphisms; T2D, type 2 diabetes.

Interestingly, two articles focused on causal risk factors for early-onset MDD (Michalek *et al.*, 2017; Sequeira *et al.*, 2017). The latter (Sequeira *et al.*, 2017) showed that genetic liability to early menarche was causally associated with higher levels of depressive symptoms at 14 years but not after 14 years. The first one (Michalek *et al.*, 2017) found that genetic predisposition to shorter telomere lengths was causally associated with increased risk for childhood-onset MDD.

### Mendelian randomization in attention-deficit and hyperactivity disorder and autism spectrum disorders

Four articles on ADHD or ASD satisfied the inclusion criteria (Jang *et al.*, 2020; Peyre *et al.*, 2021; Vilar-Ribó *et al.*, 2021; Zhao *et al.*, 2021) (Table 3). One studied the interaction between these two disorders, using a genetic instrument for ADHD and ASD (in 18 381 cases) as an outcome to show that genetic predisposition to ADHD was causally associated with an increased risk of ASD (Peyre *et al.*, 2021).

**Table 3:** Mendelian randomization in attention-deficit and hyperactivity disorder and autism spectrum disorders

Reference	Genetic instrument and exposure (*P*-value)	Main statistic test	Exclusion of pleiotropy	Outcome and findings	Outcome sample size
Zhao *et al.*, 2021	30 SNP associated with serum urate concentration (*P* < 1 × 10^−700^)	IVW	Yes	No causal association between genetic instruments for increased urate levels and ADHD risk	19 099 ADHD cases
					34 194 controls (PGC)
Peyre *et al.*, 2021	40 SNP associated with ADHD	IVW	Yes	Genetic predisposition to ADHD causally associated with an increased risk of ASD (*P* < 0.001)	18 381 ASD cases
					27 969 controls
Vilar-Ribó *et al.*, 2021	164 SNP associated with smoking initiation, alcohol or cocaine dependence, lifetime cannabis use and ever addicted to illicit drugs	IVW	No	Genetic liability to ADHD causally associated with smoking initiation (IVW OR, 1.20; *P* = 2.24 × 10^−21^), age of smoking initiation (IVW OR, 0.94; *P* = 4.27 × 10^−4^), cigarettes per day (IVW OR, 1.08; *P* = 8.23 × 10^−4^) and lifetime cannabis use (IVW OR, 1.15; *P* = 2.01 × 10^−3^)	989 ADHD cases among whom (overlapping samples):
					409 cases smoking initiation, 511 controls
					177 alcohol dependence cases, 679 controls
		MR-PRESSO			
					172 cocaine dependence cases, 777 controls
				Genetic predisposition to smoking initiation causally associated with higher ADHD risk (MR-PRESSO OR, 2.46; *P* = 3.66 × 10^−17^)	
					298 cases of lifetime cannabis use, 655 controls
				Genetic predisposition to lifetime cannabis use causally associated with higher ADHD risk (IVW OR, 1.46; *P* = 8 × 10^−4^)	345 cases of ever addicted to illicit drugs, 609 controls
	12 SNP associated with ADHD (*P* < 5 × 10^−6^)				
				No causal effect of genetic liability to ADHD on smoking cessation, alcohol dependence, cocaine dependence or ever addicted to illicit drugs and vice versa	
Jang *et al.*, 2020	341 SNP associated with substance use phenotypes (smoking, alcohol, or cannabis use)	IVW, WM, MR-Egger; Latent Causal Variable model	Yes	No causal association between genetic instruments for substance use phenotypes and the risk for ADHD	53 293 ADHD cases

ADHD, attention-deficit and hyperactivity disorder; ASD, autism spectrum disorders; IVW, inverse variance–weighted; MR-PRESSO, Mendelian Randomization Pleiotropy RESidual Sum and Outlier; OR, odds ratio; SNP, single-nucleotide polymorphism.

The second study focused on ADHD and its relationship with the use of substances (Vilar-Ribó *et al.*, 2021). It showed that being genetically exposed to ADHD causally increased the risk of smoking tobacco or cannabis and vice versa, that is genetic predisposition to smoking initiation and to lifetime cannabis was causally linked with a higher risk of suffering from ADHD. On the other side, the study did not show a causal effect of genetic liability to ADHD on smoking cessation, alcohol dependence, cocaine dependence, or addiction to illicit drugs and vice versa (Vilar-Ribó *et al.*, 2021).

### Mendelian randomization in other psychiatric disorders

We included in this section and heterogeneous group of nine articles that focused on psychiatric diseases not included in previous tables (Table 4) (Wium-Andersen *et al.*, 2015a; Jang *et al.*, 2020; Jefsen *et al.*, 2021; Jin *et al.*, 2021; Rosoff *et al.*, 2021; Vermeulen *et al.*, 2021; Zhao *et al.*, 2021; Chen *et al.*, 2022; Ni *et al.*, 2022). Among these, we included three studies on BD (Wium-Andersen *et al.*, 2016; Jefsen *et al.*, 2021; Vermeulen *et al.*, 2021). The two most recent ones focused on the relationship between cannabis or tobacco smoking and BD, finding that genetic liability to suffering from BD was causally linked with lifetime cannabis use, whereas there was no causal effect of genetic risk for lifetime cannabis use on BD risk (Jefsen *et al.*, 2021). However, genetic predisposition to smoking cigarettes appeared to be causally associated with BD risk (Vermeulen *et al.*, 2021). These studies analyzed data from the same GWAS study, including 20 352 BD cases and 31 358 controls (Stahl *et al.*, 2019). The third study explored the link between systemic inflammation and BD, finding that increased plasmatic CRP was causally associated with the development of late-onset BD, although the number of patients included in the study was limited (93 late-onset BD cases) (Wium-Andersen *et al.*, 2016).

**Table 4:** Mendelian randomization in other psychiatric disorders

Reference	Genetic instrument and exposure (*P*-value)	Main statistic test	Exclusion of pleiotropy	Outcome and findings	Outcome sample size
Chen *et al.*, 2022	2422 SNPs associated with increased 41 systemic inflammatory regulators (*P* < 1 × 10^−6^)	IVW	Yes	Genetic predisposition to increased CTAK (*P* = 1.17 × 10^−2^) and IL-18 (*P* = 1 × 10^−2^) causally associated with higher OCD risk (*P* < 5 × 10^−2^)	2688 OCD cases,
					7037 controls
				Causal association between the genetic predisposition to OCD and the levels of inflammatory regulators not investigated	
Ni *et al.*, 2022	>700 SNPs associated with gut microbiota and >3 SNPs associated with each psychiatric disorder (*P* < 1 × 10^−5^)	IVW	Yes	Genetic predisposition to increased	46 351 ASD cases, 51 710 BD, 14 307 TS, 9725 OCD cases, 23 809 AD, 18,473 controls (MiBioGen)
				Family Prevotellaceae causally associated with higher ASD risk (*P* = 5.31 × 10^−4^)	
				Class Betaproteobacteria causally associated with higher BD risk (*P* = 1.53 × 10^−3^)	
				Class Bacteroidia and order Bacteroidales with causally associated with higher TS risk (*P* = 2.51 × 10^−3^ and 2.51 × 10^−3^)	
				No causal association between genetic instruments for gut microbiota composition and the risk for OCD or AD	
				No causal association between genetic instruments for psychiatric disorders and risk of different gut microbiota composition	
Jin *et al.*, 2021	93 SNP associated with PCOS (*P* < 5 × 10^−8^)	IVW, WM	Yes	Genetic predisposition to PCOS causally associated with higher OCD risk based on IVW (OR, 1.339; 95% CI, = 1.083–1.657; *P* = 0.007) and WM analysis (OR, 1.493; 95% CI, 1.145–1.946; *P* = 0.003)	2688 OCD cases and 7037 controls (PGC)
					7016 AD cases and 14 745 controls
				No causal association between genetic instruments for PCOS predisposition and AD risk	
Zhao *et al.*, 2021	30 SNP associated with serum urate concentration (*P* < 1 × 10^−700^)	IVW	Yes	No causal association between genetic instruments for increased urate levels and the risk for the psychiatric disorders analyzed	2688 OCD cases and 7037 controls
					16 992 AN cases and 55 525 controls
					23 212 PTSD cases and 151 447 controls
Rosoff *et al.*, 2021	74 SNP associated with educational attainment (*P* < 5 × 10^−8^)	IVW	Yes	Genetically determined increased educational attainment causally associated with reduced AUD risk (OR_IVW_ = 0.508; 95% CI, 0.315–0.819; *P* = 5.52 × 10^−3^)	8485 AUD cases
					20 657 controls
Wium-Andersen *et al.*, 2015a	2 SNP associated with alcohol consumption (*P* < 1 × 10^−5^)	IVW	No	Genetic predisposition to alcohol consumption causally associated with higher risk for AUD (OR, 28.6; 95% CI, 6.47–126 for an increase of 1 drink/day estimated from the genetic instrument; *P* = NG)	4736 cases of AUD
					78 154 total participants
Jefsen *et al.*, 2021	6 SNP associated with lifetime cannabis use	IVW regression	Yes	Genetic liability to BD causally associated with lifetime cannabis use (*P* = 7 × 10^−6^)	20 352 BD cases and 31 358 controls
	17 with bipolar disorder (*P* < 5 × 10^−8^)				
				No causal effect of the genetic risk for lifetime cannabis use on the risk for BD.	
Vermeulen *et al.*, 2021	126 SNP associated with smoking cigarettes (*P* < 5 × 10^−8^)	IVW	Yes	Genetic predisposition to smoking cigarettes causally associated with BD risk (smoking initiation OR_IVW_ = 1.46; 95% CI, 1.28–1.66; *P* = 1.44 × 10−8, lifetime smoking OR_IVW_ = 1.72; 95% CI, 1.29–2.28; *P* = 1.8 × 10^−4^)	20 352 BD cases and 31 358 controls
Wium-Andersen *et al.*, 2016	4 SNP associated with elevated CRP (*P* < 5 × 10^−8^)	Instrumental variable analysis	Yes	Doubling in genetically determined CRP causally associated with late-onset BD risk (OR, 4.66; 95% CI, 0.89–24.3; *P* = 0.15)	93 late-onset BD cases and 78 809 controls

(GS)MR, (generalized summary-based) Mendelian randomization; AD, anxiety disorder; AN, anorexia nervosa; ASD, autism spectrum disorder; AUD, alcohol use disorder; BD, bipolar disorder; CI, confidence interval; CRP, c-reactive protein; CTACK, cutaneous T-cell attracting chemokine; IVW, inverse variance–weighted; OCD, obsessive-compulsive disorder; OR, odds ratio; PCOS, polycystic ovary syndrome; SNP, single-nucleotide polymorphism; TS, Tourette syndrome; WM, weighted median.

Only one study (Rosoff *et al.*, 2021) satisfied our inclusion criteria among those that employed MR in alcohol use disorder (AUD). In this work, investigators used a genetic instrument for higher educational attainment and found that it was causally associated with lower AUD risk in 8485 AUD cases (Rosoff *et al.*, 2021). Intriguingly, a recent study found that genetic predisposition to polycystic ovary syndrome was causally associated with higher obsessive-compulsive disorder (OCD) risk but not with some other psychiatric disorders (i.e. anxiety disorder and SCZ) (Jin *et al.*, 2021). Considering the inflammatory component in PCOS pathophysiology, it is very interesting to report that a recent study showed that genetic predisposition to increased inflammatory markers (CTAK and IL-18) was causally associated with higher OCD risk.

## Discussion and limitations

We systematically reviewed the evidence of the employ of MR in psychiatric diseases. We included 50 articles, divided into four groups on the basis of the psychiatric disorder assessed: SCZ (the most numerous group), MDD, ADHD/ASD, or other psychiatric diseases.

Overall, the present findings confirm that MR offers a unique opportunity of unraveling causal links in risk factors and etiological elements of psychiatric diseases, both in specific disorders and transdiagnostically.

This is especially true considering the important challenges encountered when employing more traditional research methods to ascertain causality between a certain risk factor and psychiatric disorders, which have often a multifactorial cause, and a behavioral impact that may expose the patients to further risk factors. As mentioned in the introduction, MR can control for reverse causality when investigating disease causes and can use existing GWAS studies with no need for long, time-consuming longitudinal cohort recruiting. Furthermore, the ever-increasing development of (epi)genome sequencing technologies is providing additional data for further MR studies. No study included here studied epigenomic data with MR, but this is an expanding field (Grau-Perez *et al.*, 2019) and might be an interesting perspective for psychiatric diseases.

Most commonly, studies included in this review evaluated the impact of metabolic traits, inflammation, or substance abuse on psychiatric risk. Few significant, causal links between psychiatric diseases and risk factors are confirmed by different groups in different populations, at the same time. However, some promising findings emerge. Overall, the causal relationships between depression and cardiovascular risk (of stroke in particular) that have been identified (Cai *et al.*, 2019; Gill *et al.*, 2019; Lu *et al.*, 2021) are particularly interesting considering the increasing evidence on depression and serotoninergic axis dysregulation as important players in stroke physiopathology (Colpo *et al.*, 2019; Damsbo *et al.*, 2019; Saccaro *et al.*, 2022a). While the present review did not identify a definitive causal link between systemic inflammation and psychiatric disorders (the results on SCZ, MDD, or BD patients, for example, are not conclusive), the link between inflammation and psychiatric disorders is another topic of major interest in recent years (Wium-Andersen *et al.*, 2016; Han and Ham, 2021; Murphy *et al.*, 2021; Saccaro et al., 2021a, 2021b; Saccaro *et al.*, 2022b), and further studies, employing MR as well, could unravel the pathophysiological role of inflammation in psychiatric disorders, with diagnostic, prognostic or therapeutic potential. To do so, however, it is important to focus on the right targets and to correctly interpret results. As a paradigmatic example, Perry *et al.* (2021a) and Hartwig *et al.* (2017) found that genetic variants in the IL-6 pathway purportedly linked with higher serum IL-6 or sIL-6R levels are causally associated with SCZ. While the interpretation offered by the authors is that higher levels of such plasmatic markers are causally implied in the SCZ development, a mechanistic insight into the IL-6 pathway in humans is lacking, and, thus, it has been argued that these conclusions need to be tempered (Pearce, 2022). In fact, other interpretations of these results are possible. For instance, there is evidence in animal models that maternal immune activation causes lasting proinflammatory changes, including higher IL-6 plasmatic levels, in the fetus (Mandal *et al.*, 2013). Thus, variants associated with higher serum IL-6 levels might, in fact, be associated with pregnancy complications or maternal-fetal interactions, which are well-known risk factors for SCZ (Pearce, 2022). Should this be the case, the exclusion restriction assumption (which, as discussed in the background, is crucial for MR soundness), would not be respected. Although the aforementioned findings that we have taken as an example, in this case, remain relevant and interesting, it is crucial to critically evaluate the results of MR and consider potential alternative interpretations or confounders, as recapitulated in Fig. 1.

Similarly, two independent studies (Table 4) identified causal roles of genetic predisposition to PCOS (Jin *et al.*, 2021) and higher IL-18, a proinflammatory cytokine (Chen *et al.*, 2022), in the risk of developing OCD. However, IL-18 itself has been associated with PCOS (Yang *et al.*, 2011; Zhang *et al.*, 2020), although the mechanisms underlying these associations are yet to be clarified. While the authors appropriately excluded pleiotropy in the MR analyses (Jin *et al.*, 2021; Chen *et al.*, 2022), further research is needed to ascertain the role of inflammation, PCOS, or other uninvestigated variables [such as metabolic syndrome, linking the previous two risk factors (March *et al.*, 2010)], in the pathogenesis of OCD.

Another example of cases in which key MR assumptions may not be met is the utilization of fat mass and obesity-associated protein (FTO) alleles as IV for BMI. Although FTO alleles have been used as a genetic instrument for BMI in MR studies (Tan *et al.*, 2019), evidence suggests that this might not be a reliable BMI indicator (Walter *et al.*, 2015). Should this be the case, the relevance assumption (that the variant is associated with the risk factor, or exposure, in study), would not be met.

It is, thus, clear how MR studies at the same time guide and are guided by mechanistic, preclinical ones. In fact, the choice of genetic targets is clearly of the utmost importance for performing MR studies, as the whole analysis relies on this step. In some cases, articles employed genetic instruments, whose phenotypes are more difficultly identifiable, for instance, physical activity as measured by accelerometer in MDD, which seems to be protective for MDD (Choi *et al.*, 2019). Although a relaxed threshold has been used in this study to select SNP for the genetic instrument, the findings are in agreement with existing evidence on the association between reduced physical activity and unipolar (Burton *et al.*, 2013) or bipolar depression (Saccaro *et al.*, 2021c) and actigraphy is an emerging technique, among others, for monitoring psychiatric diseases.

As mentioned above, thanks to the inclusion of results from different psychiatric disorders, this systematic review unveils transdiagnostic findings across several distinct psychiatric diseases that deserve further investigation. Besides the causal relationships between psychiatric disorders (ADHD and SCZ in particular) and smoking, which had already been identified by previous works focused on substance abuse (Treur *et al.*, 2021), we identify some preliminary transdiagnostic evidence of the causal involvement of CRP levels in the pathophysiology of late-onset BD and SCZ, that warrants additional research, as mentioned above. In fact, this link is particularly relevant in light of the increasing evidence showing that peripheral inflammation is associated with neuroinflammation and can have an impact on central nervous system development and function (Felger, 2018), besides being associated with psychiatric symptoms and disorders (Saccaro et al., 2021a, 2021b). Our transdiagnostic approach revealed also interesting causal relationships between psychiatric disorders. Genetic liability to anxiety was causally associated with higher SCZ risk (Jones *et al.*, 2020), and a DSM/ICD diagnosis of ADHD was causally associated with an increased risk of ASD (Peyre *et al.*, 2021). While observational studies had highlighted some of these associations, MR allows to speculate on the direction of causality, which may lead to new avenues of pathophysiological research and prevention.

However, the aforementioned findings are in part undermined by minor methodological issues. In fact, some caveats must be reminded, since important assumptions need to be met before considering findings from MR trustworthy, as detailed in the introduction. Some of these are inherent in MR designs, and, thus, accounted for in the included articles. These include, for example, the statistical problems derived from the fact that, in MR, the randomization process is confided to the natural distribution in the population of genetic variants, which may sometimes be, in fact, nonrandom (e.g. due to linkage disequilibrium, or ‘cryptic relatedness’), which have been discussed in detail in the MR literature (Voight and Pritchard, 2005; Nitsch *et al.*, 2006; Boef *et al.*, 2015). Furthermore, the heterogeneity of the populations included in the MR studies may represent a confounder in the analyses performed by the papers that we reviewed. Population heterogeneity can impact both the definition of the genetic IV and of the outcome population. However, our stringent inclusion criteria (exclusively DSM/ICD-based diagnoses) reduce the risk of unwanted population heterogeneity in the samples of psychiatry patients.

Other important points include taking account of potential pleiotropy, choosing solid genetic instruments, and precise inclusion/exclusion criteria for patients and control. In fact, during the full-text screening of studies for inclusion in the present systematic review, we found that a considerable number of articles enrolled some or all psychiatric patients based on self-assessment questionnaires or clinical scores other than DSM or ICD criteria. Accurate and homogenous diagnostic criteria are essential in genetic studies in particular to avoid biases (such as selection bias) and to obtain results that are meaningful to the clinical population studied. For these reasons, we included fewer studies compared to other existing reviews of MR in specific psychiatric disorders (Belbasis *et al.*, 2018; Köhler *et al.*, 2018; Treur *et al.*, 2021). For instance, the majority of studies on MDD did not use DSM nor ICD to diagnose MDD as an outcome and were therefore excluded. Similarly, many works on PTSD were based on data from the UK Biobank [which used self-reported data (Davis *et al.*, 2020), or from the PGC-Posttraumatic Stress Disorder group (PGC-PTSD) (Duncan *et al.*, 2018)], which included studies that did not diagnose patients based on DSM nor ICD (but, for example, based on a Structured Interview Scale derived from the PTSD Checklist or the modified PTSD Symptom Scale). These studies were, thus, not included. On the contrary, other disorders, for example, SCZ, were accurately diagnosed according to the DSM or the ICD in most of the papers we examined. Our stringent criteria on patients’ diagnoses allowed us to select a sample of studies that have very good scores in the NOS criteria ‘selections of patients’, in agreement with the overall fair scores in the other domains of the NOS evaluation (Supplementary eTable 2, Supplemental Digital Content 1, http://links.lww.com/PG/A287).

On the other side, the vast majority of the studies we included did accurately assess and account for possible pleiotropy in their analyses.

One last limitation common to some papers that we included (e.g. Choi *et al.*, 2019; Milaneschi *et al.*, 2019) may be the fact that researchers employed relaxed *P*-values thresholds for SNP selection to build the genetic instrument. While this method of relaxing the statistical threshold is often used in MR research when few significant SNPs are available, it exposes to the risk of basing the research on false genetic instruments or of not meeting the relevance assumption.

This review shows how MR can offer unique opportunities for unraveling causal links in risk factors and etiological elements of specific psychiatric diseases and across disorders. These relationships are otherwise difficult to uncover, but some methodological flaws in the existing literature limit the reliability of results of MR studies in psychiatry and probably underlie result heterogeneity. While most existing MR studies in psychiatry accurately employ and report analyses to exclude pleiotropy, as well as sources of genetic IVs, a few points are pivotal for future MR studies. These should first of all be informed by results from other study designs and use them to validate their findings and look for potential confounders; second, they should attempt to falsify their key assumptions, or at least clearly state them; and, finally, MR studies in psychiatry should carefully assess and report whether the patients included both for the exposure and the outcome analysis are indeed psychiatric patients with a certain DSM- or ICD-based psychiatric diagnosis.

## Acknowledgements

Figure 1 was created with Biorender.com.

PROSPERO registration: the protocol is registered in PROSPERO(CRD42021285647).

### Conflicts of interest

There are no conflicts of interest.

## Supplementary Material


