# Supplementary material for: Applications of Mendelian randomization in psychiatry: a comprehensive systematic review
Source: Psychiatr Genet. 2022 Oct 20;32(6):199–213. doi: 10.1097/YPG.0000000000000327 (PMC9648985; doi:10.1097/YPG.0000000000000327)
Supplement: Supplementary file 1 [file pg-32-199-s001.pdf]

Supplementary Information

Applications of Mendelian Randomization in Psychiatry, a systematic review

Luigi F. Saccaro, Simone Gasparini, Grazia Rutigliano

eTable1. PRISMA guidelines for systematic reviews and meta-analysis

eTable2. Risk of bias in studies included in the systematic review: Newcastle Ottawa Scale, NOS

eTable1. PRISMA guidelines for systematic reviews and meta-analysis

#	Section/topic	Checklist item and brief description of how the criteria were handled	Section, page
**TITLE**			
1	Title	*Identify the report as a systematic review, meta-analysis, or both.*<br><br>The study has been identified as a systematic review of mendelian randomization (MR) applied to psychiatric disorders	Title
**ABSTRACT**			
2	Structured summary	*Provide a structured summary including, as applicable: background; objectives; data sources; study eligibility criteria, participants and interventions; study appraisal and synthesis methods; results; limitations; conclusions and implications of key findings; systematic review registration number.*<br><br>All relevant information has been included in the abstract.	Abstract
**INTRODUCTION**			
3	Rationale	*Describe the rationale for the review in the context of what is already known.*<br><br>Psychiatric disorders are a heterogeneous group of prevalent and often severe and chronic diseases that, besides the heavy personal burden on patients and caregivers, exact a heavy socio-economic toll. It is particularly difficult to identify risk factors and causative mechanisms for psychiatric diseases due their multifactorial nature, the limited physiopathological and etiological insight we have,	Introduction

		the many confounding factors, and to the potential reverse causality between the risk factors and these diseases. These characteristics make mendelian randomization (MR) a precious tool for studying psychiatric diseases. MR is an analytical method that employs genetic variants linked to a certain risk factor, to assess if an observational association between that risk factor and a health outcome is compatible with a causal relationship.	
4	Objectives	*Provide an explicit statement of questions being addressed with reference to participants, interventions, comparisons, outcomes, and study design (PICOS).*<br><br>To systematically review the applications and findings of MR in psychiatric diseases.	Introduction
**METHODS**			
5	Protocol and registration	*Indicate if a review protocol exists, if and where it can be accessed (e.g., Web address), and, if available, provide registration information including registration number.*<br><br>The protocol is listed in the PROSPERO register (registration number: CRD42021285647).	Methods, Search strategy and selection criteria

6	Eligibility criteria	*Specify study characteristics (e.g., PICOS, length of follow-up) and report characteristics (e.g., years considered, language, publication status) used as criteria for eligibility, giving rationale.*<br><br>We included original published articles written in English, with no restrictions on publication date.<br><br>- <u>Population</u>: psychiatric patients of any age, with any psychiatric diagnosis according to DSM or ICD criteria<br>- <u>Intervention</u>: employment of MR in genetic analysis, applied to identification of risk factors, to neuroimaging correlations or to other settings relating to psychiatry (including studies that focused on how psychiatric diseases might be risk factors for other conditions<br>- <u>Comparisons</u>: presence or absence of genetic traits that are determinants of exposure to a certain risk factor (which could be a psychiatric disorder or other risk factors)<br>- <u>Outcome</u>: the risk for a certain condition (both psychiatric and not).<br>- <u>Study design</u>: all study designs apart from case reports, case series, conference abstracts and presentations, pilot/feasibility studies, reviews, meta-analyses, and systematic reviews.<br><br>Articles were excluded if they: a) were case reports, case series, conference abstracts and presentations, pilot/feasibility studies, reviews, meta-analyses, and systematic reviews; b) were written in languages other than English.	Methods, Search strategy and selection criteria
7	Information sources	*Describe all information sources (e.g., databases with dates of coverage, contact with study authors to identify additional studies) in the search and date last searched.*<br><br>We used a two-step approach. First, we searched the Web of KnowledgeSM database by Thomas Reuters ® (including Web of Science Core Collection, BIOSIS Citation Index, KCI - Korean Journal Database, MEDLINE, Russian Science Citation Index, and SciELO Citation Index), and Scopus. Secondly, we performed an electronic manual search	Methods, Search strategy and selection criteria

		of the lists of the references of retrieved articles. Duplicate references were manually excluded.	
8	Search	*Present full electronic search strategy for at least one database, including any limits used, such that it could be repeated.*<br><br>The search strategy included terms related to MR and psychiatry [((Mendelian randomization) OR (Mendelian randomisation)) AND (psychiatry OR bipolar disorder OR borderline personality disorder OR schizophrenia OR depression OR ADHD OR anxiety OR PTSD OR panic)) NOT (Review OR metanalysis OR meta-analysis))] for articles published until 17/10/2021.	Methods, Search strategy and selection criteria
9	Study selection	*State the process for selecting studies (i.e., screening, eligibility, included in systematic review, and, if applicable, included in the meta-analysis).*<br><br>The identified articles were screened by title and abstract, and the full text of surviving articles were further inspected for eligibility against *a priori* defined inclusion and exclusion criteria.	Methods, Search strategy and selection criteria, Figure 1
10	Data collection process	*Describe method of data extraction from reports (e.g., piloted forms, independently, in duplicate) and any processes for obtaining and confirming data from investigators.*<br><br>Data extraction was performed by independent researchers (LFS, SG). Any discrepancy was discussed until a consensus was reached. Disagreements were resolved by a third reviewer (GR).	Methods, Data extraction

11	Data items	*List and define all variables for which data were sought (e.g., PICOS, funding sources) and any assumptions and simplifications made.*<br><br>The following variables were extracted from each article: authors and year of publication, sample size, genetic information analyzed, main MR method, main findings, presence of pleiotropy analysis, psychiatric disorder considered as an outcome.	Methods, Data extraction
12	Risk of bias in individual studies	*Describe methods used for assessing risk of bias of individual studies (including specification of whether this was done at the study or outcome level), and how this information is to be used in any data synthesis.*<br><br>The quality of the selected studies was assessed independently by two reviewers (SG, LG) with the Newcastle-Ottawa Scale (NOS).	Methods, Risk of bias
13	Summary measures	*State the principal summary measures (e.g., risk ratio, difference in means).*<br><br>The main findings of the individual studies were reported in Tables 1-5	Tables 1-5
14	Synthesis of results	*Describe the methods of handling data and combining results of studies, if done, including measures of consistency (e.g., I^2) for each meta-analysis.*<br><br>The main findings of the individual studies were reported in tables 1-5. Data from individual studies could not be combined due to high heterogeneity.	Tables 1-5

15	Risk of bias across studies	*Specify any assessment of risk of bias that may affect the cumulative evidence (e.g., publication bias, selective reporting within studies).*<br><br>The risk of bias and concerns regarding applicability were analyzed for each domain of the NOS.	Methods, Data analysis
16	Additional analyses	*Describe methods of additional analyses (e.g., sensitivity or subgroup analyses, metaregression), if done, indicating which were pre-specified.*<br><br>N/A	N/A
**RESULTS**			
17	Study selection	*Give numbers of studies screened, assessed for eligibility, and included in the review, with reasons for exclusions at each stage, ideally with a flow diagram.*<br><br>All details are depicted in the PRISMA flow-chart (Figure 2), and described in the main text.	Results; Figure 2
18	Study characteristics	*For each study, present characteristics for which data were extracted (e.g., study size, PICOS, follow-up period) and provide the citations.*<br><br>For included studies, characteristics and citations are listed in Tables 1-5.	Results; Tables 1-5
19	Risk of bias within studies	*Present data on risk of bias of each study and, if available, any outcome level assessment (see item 12).*<br><br>Risk of bias is reported in the main text. Details are reported in Supplementary material (eTable 2).	Results, Quality assessment; Supplementary material, eTable 2

20	Results of individual studies	*For all outcomes considered (benefits or harms), present, for each study: (a) simple summary data for each intervention group (b) effect estimates and confidence intervals, ideally with a forest plot.*<br><br>N/A (no summary data due to high heterogeneity). The main findings of the individual studies are summarized in main text and reported in Tables 1-5.	Results; Tables 1-5
21	Synthesis of results	*Present results of each meta-analysis done, including confidence intervals and measures of consistency.*<br><br>N/A	N/A
22	Risk of bias across studies	*Present results of any assessment of risk of bias across studies (see Item 15).*<br><br>Risk of bias is reported in the main text. Details are reported in Supplementary material (eTable 2).	Results, Quality assessment; Supplementary material, eTable 2
23	Additional analysis	*Give results of additional analyses, if done (e.g., sensitivity or subgroup analyses, meta-regression [see Item 16]).*<br><br>N/A	N/A
**DISCUSSION**			
24	Summary of evidence	*Summarize the main findings including the strength of evidence for each main outcome; consider their relevance to key groups (e.g., healthcare providers, users, and policy makers).*	Discussion
25	Limitations	*Discuss limitations at study and outcome level (e.g., risk of bias), and at review-level (e.g., incomplete retrieval of identified research, reporting bias).*	Discussion

26	Conclusions	*Provide a general interpretation of the results in the context of other evidence, and implications for future research.*	Discussion
**FUNDING**			
27	Funding	*Describe sources of funding for the systematic review and other support (e.g., supply of data); role of funders for the systematic review.*<br><br>N/A.	N/A

REFERENCE	SELECTIVITY	COMPARABILITY	OUTCOME	FINAL SCORE
Andreu-Bernabeu, Á. et al., 2022. ‘Polygenic contribution to the relationship of loneliness and social isolation with schizophrenia’, Nature Communications. Springer US, 13(1). doi: 10.1038/s41467-021-27598-6.	4	2	1	**7**
Arafat, S., Minica, C.C., 2018. Fetal origins of mental disorders? an answer based on mendelian randomization. Twin Res. Hum. Genet. 21, 485–494. https://doi.org/10.1017/thg.2018.65	4	2	3	**9**
Byrne, E.M., Ferreira, M.A.R., Xue, A., Lindström, S., Jiang, X., Yang, J., Easton, D.F., Wray, N.R., ChenevixTrench, G., 2019. Is Schizophrenia a Risk Factor for Breast Cancer?-Evidence from Genetic Data. Schizophr. Bull. 45, 1251–1256. https://doi.org/10.1093/schbul/sby162	4	2	2	**8**
Cai, H., Cai, B., Zhang, H., Sun, W., Wang, Y., Zhou, S., Ye, Z., Zhang, Z., Liang, J., 2019. Major depression and small vessel stroke: a Mendelian randomization analysis. J. Neurol. 266, 2859–2866. https://doi.org/10.1007/s00415-019-09511-w	4	2	1	**7**
Chen, J., Chen, R., Xiang, S., Li, N., Gao, C., Wu, C., Zhang, Q., Zhao, Y., Liao, Y., Stewart, R., Xu, Y., Shi, Y., Li, Z., 2021. Cigarette smoking and schizophrenia: Mendelian randomisation study. Br. J. Psychiatry 218, 98–103. https://doi.org/10.1192/bjp.2020.116	4	1	1	**6**
Chen, X. et al., 2022. ‘Systemic inflammatory regulators and 7 major psychiatric disorders: A two-sample Mendelian randomization study’, Progress in Neuro-Psychopharmacology and Biological Psychiatry. Elsevier Inc., 116(13), p. 110534. doi: 10.1016/j.pnpbp.2022.110534.	4	1	1	**6**
Choi, K.W., Chen, C.Y., Stein, M.B., Klimentidis, Y.C., Wang, M.J., Koenen, K.C., Smoller, J.W., Wray, N.R., Ripke, S., Mattheisen, M., Trzaskowski, M., Byrne, E.M., Abdellaoui, A., Adams, , M.M., Werge, T., Lewis, C.M., Levinson, D.F., Breen, G., Børglum, A.D., Sullivan, P.F., 2019. Assessment of bidirectional relationships between physical activity and depression among adults a 2-sample Mendelian randomization study. JAMA Psychiatry 76, 399–408. https://doi.org/10.1001/jamapsychiatry.2018.4175	4	2	1	**7**
Clarke, T.K., Obsteter, J., Hall, L.S., Hayward, C., Thomson, P.A., Smith, B.H., Padmanabhan, S., Hocking, L.J., Deary, I.J., Porteous, D.J., McIntosh, A.M., 2017. Investigating shared aetiology between type 2 diabetes and major depressive disorder in a population based cohort. Am. J. Med. Genet. Part B Neuropsychiatr. Genet. 174, 227–234. https://doi.org/10.1002/ajmg.b.32478	4	1	1	**6**

Gage, S.H., Jones, H.J., Burgess, S., Bowden, J., Davey Smith, G., Zammit, S., Munafò, M.R., 2017a. Assessing causality in associations between cannabis use and schizophrenia risk: A two-sample Mendelian randomization study. Psychol. Med. 47, 971–980. https://doi.org/10.1017/S0033291716003172	4	1	1	**6**
Gage, S.H., Jones, H.J., Taylor, A.E., Burgess, S., Zammit, S., Munafò, M.R., 2017b. Investigating causality in associations between smoking initiation and schizophrenia using Mendelian randomization. Sci. Rep. 7, 1–8. https://doi.org/10.1038/srep40653	4	1	1	**6**
Gao, X., Meng, L.X., Ma, K.L., Liang, J., Wang, H., Gao, Q., Wang, T., 2019. The bidirectional causal relationships of insomnia with five major psychiatric disorders: A Mendelian randomization study. Eur. Psychiatry 60, 79–85. https://doi.org/10.1016/j.eurpsy.2019.05.004	4	2	1	**7**
Gill, D., James, N.E., Monori, G., Lorentzen, E., Fernandez-Cadenas, I., Lemmens, R., Thijs, V., Rost, N.S., Scott, R., Hankey, G.J., Lindgren, A., Jern, C., Maguire, J.M., 2019. Genetically Determined Risk of Depression and Functional Outcome after Ischemic Stroke: Mendelian Randomization Study. Stroke 50, 2219–2222. https://doi.org/10.1161/STROKEAHA.119.026089	4	1	1	**6**
Hartwig, F.P., Borges, M.C., Horta, B.L., Bowden, J., Davey Smith, G., 2017. Inflammatory biomarkers and risk of schizophrenia: A 2-sample mendelian randomization study. JAMA Psychiatry 74, 1226–1233. https://doi.org/10.1001/jamapsychiatry.2017.3191	4	1	1	**6**
Huang, J., Zuber, V., Matthews, P.M., Elliott, P., Tzoulaki, J., Dehghan, A., 2020. Sleep, major depressive disorder, and Alzheimer disease: A Mendelian randomization study. Neurology 95, e1963–e1970. https://doi.org/10.1212/WNL.0000000000010463	4	2	1	**7**
Hung, C.F., Rivera, M., Craddock, N., Owen, M.J., Gill, M., Korszun, A., Maier, W., Mors, O., Preisig, M., Rice, J.P., Rietschel, M., Jones, L., Middleton, L., Aitchison, K.J., Davis, O.S.P., Breen, G., Lewis, C., Farmer, A., McGuffin, P., 2014. Relationship between obesity and the risk of clinically significant depression: Mendelian randomisation study. Br. J. Psychiatry 205, 24–28. https://doi.org/10.1192/bjp.bp.113.130419	4	1	1	**6**
Inoshita, M., Numata, S., Tajima, A., Kinoshita, M., Umehara, H., Nakataki, M., Ikeda, M., Maruyama, S., Yamamori, H., Kanazawa, T., Shimodera, S., Hashimoto, R., Imoto, I., Yoneda, H., Iwata, N., Ohmori, T., 2016. A significant causal association between C-reactive protein levels and schizophrenia. Sci. Rep. 6, 1–8. https://doi.org/10.1038/srep26105	4	1	1	**6**

Jang, S.K., Saunders, G., Liu, M., Jiang, Y., Liu, D.J., Vrieze, S., 2020. Genetic correlation, pleiotropy, and causal associations between substance use and psychiatric disorder. Psychol. Med. https://doi.org/10.1017/S003329172000272X	4	2	1	**7**
Jefsen, O.H., Speed, M., Speed, D., Østergaard, S.D., 2021. Bipolar disorder and cannabis use: A bidirectional two-sample Mendelian randomization study. Addict. Biol. 1–7. https://doi.org/10.1111/adb.13030	4	1	1	**6**
Jin, L., Yu, J., Chen, Y., Pang, H., Sheng, J., Huang, H., 2021. Polycystic Ovary Syndrome and Risk of Five Common Psychiatric Disorders Among European Women: A Two-Sample Mendelian Randomization Study. Front. Genet. 12, 1–10. https://doi.org/10.3389/fgene.2021.689897	4	2	1	**7**
Jones, H.J., Martin, D., Lewis, S.J., Davey Smith, G., O'Donovan, M.C., Owen, M.J., Walters, J.T.R., Zammit, S., 2020. A Mendelian randomization study of the causal association between anxiety phenotypes and schizophrenia. Am. J. Med. Genet. Part B Neuropsychiatr. Genet. 183, 360–369. https://doi.org/10.1002/ajmg.b.32808	4	2	1	**7**
Johnson, Emma C.; Hatoum, Alexander S.; Deak, Joseph D.; Polimanti, Renato; Murray, Robin M.; Edenberg, Howard J.; Gelernter, Joel; Di Forti, Marta; Agrawal, Arpana. The relationship between cannabis and schizophrenia: a genetically informed perspective	4	2	1	**7**
Kim, S., Kim, K., Myung, W., Lee, H., Kim, H., Kim, D.K., Won, H.-H., 2020. Two-sample Mendelian randomization study for schizophrenia and breast cancer. Precis. Futur. Med. 4, 21–30. https://doi.org/10.23838/pfm.2019.00093	4	2	1	**7**
Kwok, M.K., Leung, G.M., Schooling, C.M., 2016. Habitual coffee consumption and risk of type 2 diabetes, ischemic heart disease, depression and Alzheimer's disease: A Mendelian randomization study. Sci. Rep. 6, 1–9. https://doi.org/10.1038/srep36500	4	1	1	**6**
Li, Z., Chen, P., Chen, J., Xu, Y., Wang, Q., Li, X., Li, C., He, L., Shi, Y., 2018. Glucose and Insulin-Related Traits, Type 2 Diabetes and Risk of Schizophrenia: A Mendelian Randomization Study. EBioMedicine 34, 182– 188. https://doi.org/10.1016/j.ebiom.2018.07.037	4	2	1	**7**
Lu, Y., Wang, Z., Georgakis, M.K., Lin, H., Zheng, L., 2021. Genetic liability to depression and risk of coronary artery disease, myocardial infarction, and other cardiovascular outcomes. J. Am. Heart Assoc. 10, 1–8. https://doi.org/10.1161/JAHA.120.017986	4	1	1	**6**
Luo, Q., Wen, Z., Li, Y., Chen, Z., Long, X., Bai, Y., Huang, S., Yan, Y., Lin, R., Mo, Z., 2020. Assessment causality in associations between serum uric acid and risk of	4	2	1	**7**

schizophrenia: A two-sample bidirectional mendelian randomization study. Clin. Epidemiol. 12, 223–233. https://doi.org/10.2147/CLEP.S236885				
Maharjan, D.T., Syed, A.A.S., Lin, G.N., Ying, W., 2021. Testosterone in Female Depression: A Meta-Analysis and Mendelian Randomization Study. Biomolecules 11, 1–11. https://doi.org/10.3390/biom11030409	4	1	1	**6**
Michalek, J.E., Kepa, A., Vincent, J., Frissa, S., Goodwin, L., Hotopf, M., Hatch, S.L., Breen, G., Powell, T.R., 2017. Genetic predisposition to advanced biological ageing increases risk for childhood-onset recurrent major depressive disorder in a large UK sample. J. Affect. Disord. 213, 207–213. https://doi.org/10.1016/j.jad.2017.01.017	4	1	1	**6**
Milaneschi, Y., Peyrot, W.J., Nivard, M.G., Mbarek, H., Boomsma, D.I., W.J.H. Penninx, B., 2019. A role for vitamin D and omega-3 fatty acids in major depression? An exploration using genomics. Transl. Psychiatry 9. https://doi.org/10.1038/s41398-019-0554-y	4	1	1	**6**
Ni, J. J. et al., 2022. 'Gut Microbiota and Psychiatric Disorders: A Two-Sample Mendelian Randomization Study', Frontiers in Microbiology, 12(February), pp. 1–11. doi: 10.3389/fmicb.2021.737197.	4	1	1	**6**
Pasman, J.A., Verweij, K.J.H., Gerring, Z., Stringer, S., Sanchez-roige, S., Treur, J.L., Abdellaoui, A., Nivard, M.G., Baselmans, B.M.L., Ong, J., Ip, H.F., Zee, M.D. Van Der, Bartels, M., Day, F.R., Fontanillas, P., Elson, S.L., Wit, H. De, Davis, L.K., Mackillop, J., Substance, T., Disorders, U., Group, W., Kaprio, J., Boks, M.P.M., Bell, J.T., Spector, T.D., Gelernter, J., Boomsma, D.I., Martin, N.G., Macgregor, S., Perry, J.R.B., Palmer, A.A., Posthuma, D., Munafò, M.R., Gillespie, N.A., Derks, E.M., Vink, J.M., 2018. GWAS of lifetime cannabis use reveals new risk loci, genetic overlap with psychiatric traits, and a causal influence of schizophrenia. Nat.Neurosci. 21.	4	1	3	**8**
Perry, B.I., Burgess, S., Jones, H.J., Zammit, S., Upthegrove, R., Mason, A.M., Day, F.R., Langenberg, C., Wareham, N.J., Jones, P.B., Khandaker, G.M., 2021. The potential shared role of inflammation in insulin resistance and schizophrenia: A bidirectional two-sample mendelian randomization study. PLoS Med. 18, 1–21. https://doi.org/10.1371/JOURNAL.PMED.1003455	4	1	1	**6**
Peters, T., Nüllig, L., Antel, J., Naaresh, R., Laabs, B.H., Tegeler, L., Amhaouach, C., Libuda, L., Hinney, A., Hebebrand, J., 2020. The Role of Genetic Variation of BMI, Body Composition, and Fat Distribution for Mental Traits and Disorders: A Look-Up and Mendelian Randomization Study. Front. Genet. 11. https://doi.org/10.3389/fgene.2020.00373	4	1	1	**6**

Peyre, H., Schoeler, T., Liu, C., Hoertel, N., Havdahl, A., 2021. Combining multivariate genomic approaches to elucidate the comorbidity between autism spectrum disorder and attention deficit hyperactivity disorder. https://doi.org/10.1111/jcpp.13479	4	1	1	**6**
Polimanti, R., Gelernter, J., Stein, D.J., 2018. Genetically determined schizophrenia is not associated with impaired glucose homeostasis. Schizophr. Res. 195, 286–289. https://doi.org/10.1016/j.schres.2017.10.033	4	2	1	**7**
Prins, B.P., Abbasi, A., Wong, A., Vaez, A., Nolte, I., Franceschini, N., Stuart, P.E., Guterriez Achury, J., Mistry, V., Bradfield, J.P., Valdes, A.M., Bras, J., Shatunov, A., Lu, C., Han, B., Raychaudhuri, S., Bevan, S., Mayes, M.D., Tsoi, L.C., Evangelou, E., Nair, R.P., Grant, S.F.A., Polychronakos, C., Radstake, T.R.D., van Heel, D.A., Dunstan, M.L., Wood, N.W., Al-Chalabi, A., Dehghan, A., Hakonarson, H., Markus, H.S., Elder, J.T., Knight, J., Arking, D.E., Spector, T.D., Koeleman, B.P.C., van Duijn, C.M., Martin, J., Morris, A.P., Weersma, R.K., Wijmenga, C., Munroe, P.B., Perry, J.R.B., Pouget, J.G., Jamshidi, Y., Snieder, H., Alizadeh, B.Z., 2016. Investigating the Causal Relationship of C-Reactive Protein with 32 Complex Somatic and Psychiatric Outcomes: A Large-Scale Cross-Consortium Mendelian Randomization Study. PLoS Med. 13, 1–29. https://doi.org/10.1371/journal.pmed.1001976	4	1	2	**7**
Rosoff, D.B., Clarke, T.K., Adams, M.J., McIntosh, A.M., Davey Smith, G., Jung, J., Lohoff, F.W., 2021. Educational attainment impacts drinking behaviors and risk for alcohol dependence: results from a two-sample Mendelian randomization study with ~780,000 participants. Mol. Psychiatry 26, 1119–1132. https://doi.org/10.1038/s41380-019-0535-9	4	1	1	**6**
Sequeira, M.E., Lewis, S.J., Bonilla, C., Smith, G.D., Joinson, C., 2017. Association of timing of menarche with depressive symptoms and depression in adolescence: Mendelian randomisation study. Br. J. Psychiatry 210, 39–46. https://doi.org/10.1192/bjp.bp.115.168617	4	0	1	**5**
Song, W., Qian, W., Wang, W., Yu, S., Lin, G.N., 2021. Mendelian randomization studies of brain MRI yield insights into the pathogenesis of neuropsychiatric disorders 1–11.	4	2	1	**7**
Taylor, A.E., Burgess, S., Ware, J.J., Gage, S.H., Richards, J.B., Davey Smith, G., Munafò, M.R., 2016. Investigating causality in the association between 25(OH)D and schizophrenia. Sci. Rep. 6, 1–9. https://doi.org/10.1038/srep26496	4	2	1	**7**
Tomioka, Y., Numata, S., Kinoshita, M., Umehara, H., Watanabe, S. ya, Nakataki, M., Iwayama, Y., Toyota, T., Ikeda, M., Yamamori, H., Shimodera, S., Tajima, A.,	4	2	1	**7**

Hashimoto, R., Iwata, N., Yoshikawa, T., Ohmori, T., 2018. Decreased serum pyridoxal levels in schizophrenia: Meta-analysis and Mendelian randomization analysis. J. Psychiatry Neurosci. 43, 194–200. https://doi.org/10.1503/jpn.170053				
Vermeulen, J.M., Wootton, R.E., Treur, J.L., Sallis, H.M., Jones, H.J., Zammit, S., Van Den Brink, W., Goodwin, G.M., De Haan, L., Munafò, M.R., 2021. Smoking and the risk for bipolar disorder: Evidence from a bidirectional Mendelian randomisation study. Br. J. Psychiatry 218, 88–94. https://doi.org/10.1192/bjp.2019.202	4	1	1	**6**
Vilar-Ribó, L., Sánchez-Mora, C., Rovira, P., Richarte, V., Corrales, M., Fadeuilhe, C., Arribas, L., Casas, M.,Ramos-Quiroga, J.A., Ribasés, M., Soler Artigas, M., 2021. Genetic overlap and causality between substance use disorder and attention-deficit and hyperactivity disorder. Am. J. Med. Genet. Part B Neuropsychiatr. Genet. 186, 140–150. https://doi.org/10.1002/ajmg.b.32827	4	1	1	**6**
Wium-Andersen, M.K., Oørsted, D.D., Nordestgaard, B.G., 2014. Elevated C-reactive protein, depression, somatic diseases, and all-cause mortality: A mendelian randomization study. Biol. Psychiatry 76, 249–257. https://doi.org/10.1016/j.biopsych.2013.10.009	4	1	3	**8**
Wium-Andersen, M.K., Ørsted, D.D., Nordestgaard, B.G., 2015a. Tobacco smoking is causally associated with antipsychotic medication use and schizophrenia, but not with antidepressant medication use or depression. Int. J. Epidemiol. 44, 566–577. https://doi.org/10.1093/ije/dyv090	4	2	3	**9**
Wium-Andersen, M.K., Ørsted, D.D., Nordestgaard, B.G., 2016. Elevated C-reactive protein and late-onset bipolar disorder in 78 809 individuals from the general population. Br. J. Psychiatry 208, 138–145. https://doi.org/10.1192/bjp.bp.114.150870	4	2	3	**9**
Wium-Andersen, M.K., Ørsted, D.D., Tolstrup, J.S., Nordestgaard, B.G., 2015b. Increased alcohol consumption as a cause of alcoholism, without similar evidence for depression: A Mendelian randomization study. Int. J. Epidemiol. 44, 526–539. https://doi.org/10.1093/ije/dyu220	4	2	3	**9**
Wootton, R.E., Richmond, R.C., Stuijfzand, B.G., Lawn, R.B., Sallis, H.M., Taylor, G.M.J., Hemani, G., Jones, H.J., Zammit, S., Davey Smith, G., Munafò, M.R., 2020. Evidence for causal effects of lifetime smoking on risk for depression and schizophrenia: A Mendelian randomisation study. Psychol. Med. 50, 2435–2443. https://doi.org/10.1017/S0033291719002678	4	2	1	**7**
Zhao, S.S., Qian, Y., Mackie, S.L., Wen, C., Mao, Y., 2021. Genetically predicted serum urate levels have no causal role on depression or other psychiatric disorders. Clin. Rheumatol. https://doi.org/10.1007/s10067- 021-05718-3	4	2	1	**7**

Zhuang, Z., Yang, R., Wang, W., Qi, L., Huang, T., 2020. Associations between gut microbiota and Alzheimer's disease, major depressive disorder, and schizophrenia. J. Neuroinflammation. https://doi.org/10.1186/s12974- 020-01961-8	4	2	1	**7**

eTable2. Risk of bias in studies included in the systematic review: Newcastle Ottawa Scale (NOS)
